# Non-extensitivity and criticality of atomic hydropathicity around a voltage-gated sodium channel’s pore: a modeling study

**DOI:** 10.1007/s10867-021-09565-w

**Published:** 2021-03-18

**Authors:** Markos N. Xenakis, Dimos Kapetis, Yang Yang, Jordi Heijman, Stephen G. Waxman, Giuseppe Lauria, Catharina G. Faber, Hubert J. Smeets, Patrick J. Lindsey, Ronald L. Westra

**Affiliations:** 1 Department of Toxicogenomics, Section Clinical Genomics, Maastricht University, PO Box 616, 6200 MD Maastricht, The Netherlands; 2 Research School for Mental Health and Neuroscience (MHeNS), Maastricht University, PO Box 616, 6200 MD Maastricht, The Netherlands; 3 Neuroalgology Unit, Fondazione IRCCS Istituto Neurologico “Carlo Besta”, via Celoria 11, 20133 Milan, Italy; 4 Department of Medicinal Chemistry and Molecular Pharmacology, Purdue University College of Pharmacy, West Lafayette, IN 47907 USA; 5 Purdue Institute for Integrative Neuroscience, West Lafayette, IN 47907 USA; 6 Department of Cardiology, CARIM School for Cardiovascular Diseases, Maastricht University, PO Box 616, 6200 MD Maastricht, The Netherlands; 7 Department of Neurology and Center for Neuroscience and Regeneration Research, Yale University School of Medicine, New Haven, CT 06510 USA; 8 Rehabilitation Research Center, Veterans Affairs Connecticut Healthcare System, West Haven, CT 06516 USA; 9 Department of Biomedical and Clinical Sciences “Luigi Sacco”, University of Milan, via G.B. Grassi 74, 20157 Milan, Italy; 10 Department of Neurology, Maastricht University Medical Center, PO Box 5800, 6202 AZ Maastricht, The Netherlands; 11 Research School for Oncology and Developmental Biology (GROW), Maastricht University, PO Box 616, 6200 MD Maastricht, The Netherlands; 12 Department of Data Science and Knowledge Engineering, Maastricht University, PO Box 616, 6200 MD Maastricht, The Netherlands

**Keywords:** Voltage-gated sodium channels, Scaling, Hydropathicity, NavAb, Non-extensitivity, Criticality

## Abstract

Voltage-gated sodium channels (NavChs) are pore-forming membrane proteins that regulate the transport of sodium ions through the cell membrane. Understanding the structure and function of NavChs is of major biophysical, as well as clinical, importance given their key role in cellular pathophysiology. In this work, we provide a computational framework for modeling system-size-dependent, i.e., cumulative, atomic properties around a NavCh’s pore. We illustrate our methodologies on the bacterial NavAb channel captured in a closed-pore state where we demonstrate that the atomic environment around its pore exhibits a bi-phasic spatial organization dictated by the structural separation of the pore domains (PDs) from the voltage-sensing domains (VSDs). Accordingly, a mathematical model describing packing of atoms around NavAb’s pore is constructed that allows—under certain conservation conditions—for a power-law approximation of the cumulative hydropathic dipole field effect acting along NavAb’s pore. This verified the non-extensitivity hypothesis for the closed-pore NavAb channel and revealed a long-range hydropathic interactions law regulating atom-packing around the NavAb’s selectivity filter. Our model predicts a PDs-VSDs coupling energy of $\sim \!282.1$ kcal/mol corresponding to a global maximum of the atom-packing energy profile. Crucially, we demonstrate for the first time how critical phenomena can emerge in a single-channel structure as a consequence of the non-extensive character of its atomic porous environment.

**Supplementary Information:**

The online version contains supplementary material available at 10.1007/s10867-021-09565-w.

## Introduction

The main function of voltage-gated sodium channels (NavChs) is to control initiation and propagation of electrical signals along neuronal tissues [1]. Enhancing our mechanistic understanding of NavCh physics is important not only for biology and protein science but also for medicine. In humans, mutations in NavCh-encoding genes can perturb structural and functional properties of the expressed protein, thus leading to abnormal electrical cellular signaling. These abnormalities may sum up and manifest at an organism level as ion channel diseases known as channelopathies [2]. Numerous studies have shown that mutations in the SCN9A gene encoding the Nav1.7 channel are causally related to inherited forms of chronic pain [3–18], cardiac arrhythmias [19, 20], and epilepsy [21].

The structural motif of a NavCh is that of a tetramer where each of the four subunits comprises a pore domain (PD) interlinked with a voltage-sensing domain (VSD). This conserved architecture facilitates a central pore through which sodium ions are conducted with high specificity. Barriers imposed to ion transport through the pore stem from the energy required for dehydrating them, i.e., removing water molecules from their hydration shell [22–24]. Dehydration of ions is achieved via interaction with the pore walls; ions have to fit closely in the pore’s confined geometry so that removed waters are immediately replaced by waters provided by the walls [22]. It is hence the interplay of geometrical and hydropathic (i.e., hydrophobic or hydrophilic) characteristics along a NavCh’s pore that determines the pore’s gating behavior [25–30], i.e., its permeability to specific ions. For example, in the case of the NavAb bacterial channel [31], a hydrophobic and wide cavity favors rapid diffusion of hydrated ions while a hydrophilic and narrow pore region acts as a selectivity filter (SF) by binding with and partially dehydrating ions. The crucial role of hydropathic effects is also manifested at scales larger than these of the pore’s microenvironment as they contribute to a NavCh’s stability (e.g., via formation of interaction networks between pore-lining structures and their surroundings [32, 33]). However, despite the plethora of NavCh structures available, modeling the synergy of hydropathic effects across different NavCh scales remains an open problem. This is due to the spatial complexity underlying hydropathic interactions [34, 35] posing significant challenges in development of suitable theoretical vehicles and computational tools for multiscale hydropathic analysis of protein structures.

Non-extensive systems [36] are characterized by thermodynamic functions that do not scale in a linear fashion with increasing system size. A hallmark of non-extensitivity is the emergence of power-law scaling reflecting the long-range nature of interactions among system components. This phenomenon is termed scale invariance (for a review, see [37]) and may be attributed to system dynamics operating within a self-organized criticality [38] regime. In this study, we test the hypothesis of non-extensitivity for the hydropathic property of a NavCh protein system based on model-retrieved atom-packing characteristics. Prior knowledge supporting this hypothesis is (a) hydropathic effects are long-range with underlying interactions laws varying across different scales [40]; (b) packing of amino acids in protein structures exhibits hydropathically driven self-similar behaviors [39]; (c) spatial inhomogeneities of the hydropathic atomic mixture around a NavCh’s pore can generate non-random patterns and topologies [41]. Accordingly, we present here a computational framework for modeling cumulative hydropathic characteristics of the atomic environment around a NavCh’s pore. For that, we utilize the finite-size scaling analysis methodologies presented in [41] and embed them into a thermodynamic context. In computational practice, the non-extensitivity hypothesis for a single NavCh structure can then be tested by investigating whether inter-atomic hydropathic interactions around the pore exhibit scale invariance or not.

In a continuation of [41], we employ the pre-open NavAb channel [31] corresponding to a closed-pore conformation. Focusing on the homotetrameric NavAb channel provides insight into a prototype (in terms of its placement in the evolutionary lineage [42, 43], as well as, architecture [31]) NavCh protein system and, hence, a basis for investigating more complex channel structures such as mammalian heteromeric NavChs. We first show that packing of atoms around its pore can be accurately described in terms of the Richards model [44] that is known to admit an algebraic *q*-generalization [45]. This led to the discovery of inflection points matching closely the conserved crossover from the PD-forming S5-S6 helices to the S1–S4 segments responsible for voltage-sensing (the structural separation of the PDs from the VSDs is a well-conserved feature of the voltage-gated ion channels superfamily [46, 47]). Thermodynamic properties of the pore were then investigated in terms of the hydropathic imbalance effect [48]. For that, we analyzed the pre- and post-inflection scaling behaviors of a hydropathic imbalance pore function [41] inferring about system-size-dependent changes in inter-atomic hydropathic interactions. This yielded a bi-phasic and scale-invariant hydropathic interactions model applying with high accuracy to the extracellular side of the NavAb’s SF. Although hydropathic scaling mechanisms in merged structural protein data have been previously investigated and claims for criticality have been made (for example see [49–51]), our work provides some first insight on how these phenomena emerge in a single NavCh protein system and might be contributing to energetic optimization of its functional architecture.

## Methods

### 3D structure preparation

We employed the pre-open I217C NavAb structure (PDB code: 3RVY), providing a snapshot of a closed-pore NavAb conformation at 2.7Åresolution. Protonation of the structure was performed with the WHAT IF software [52, 53] and the principal axes of the protonated structure were estimated with the VMD software [54]. Next, the protonated structure was embedded into a coordinate system $(\hat {\mathbf {x}},\hat {\mathbf {y}},\hat {\mathbf {z}})$ with origin *O* so that its principal pore axis, i.e., the axis approximating the direction of the channel’s pore, was aligned with the *z*-axis with orientation from the extracellular side (ES) to the intracellular side (IS) with respect to $\hat {\mathbf {z}}$. The molecular mass center $\boldsymbol {\mathrm {e}} = \frac {1}{M}{\sum }_{i=1}^{N_{c}}m_{i}\!\cdot \!\boldsymbol {\mathrm {c}}_{i}$ of the structure was set to coincide with *O*, where ***c***_*i*_ = (c_*x*,*i*_,c_*y*,*i*_,c_*z*,*i*_) is the atomic center of the *i*-th atom, *m*_*i*_ is the mass of the *i*-th atom, *N*_*c*_ = 14776 is the total number of atoms, and $M = {\sum }_{i=1}^{N_{c}}m_{i}$ is the total molecular mass. An illustration of the pre-open NavAb structural model is presented in Supplementary Information (SI), Section S1.

### Geometrical characteristics of the pore

Let us consider *P* to represent the principal pore axis with ***p*** = (p_*x*_,p_*y*_,p_*z*_) ∈ *Q* ⊂ *P* being a pore point (see SI, Section S2 for construction of *Q*). Then, the pore radius at ***p*** is given by [55]

m1 $$  R(\boldsymbol{\mathrm{p}}) = \underset{i=1,2,..,N_{c}}{min}\{||\boldsymbol{\mathrm{c}}_{i} -\boldsymbol{\mathrm{p}}|| - vdW_{i}\} $$

where || ⋅ || is the Euclidean norm and *vdW*_*i*_ is the van der Waals radius of the *i*-th atom (see Suppl. Table S1 in [41]) and the distance between ***p*** and its nearest neighbor atom corresponds to

m2 $$  D(\boldsymbol{\mathrm{p}}) = \underset{i=1,2,..,N_{c}}{min}\{||\boldsymbol{\mathrm{c}}_{i} -\boldsymbol{\mathrm{p}}||\} $$

Moreover, the outer surface radius at ***p*** is given by [41]

m3 $$  L(\boldsymbol{\mathrm{p}}) = \underset{i=1,2,..,N_{c}}{max}\{||\boldsymbol{\mathrm{c}}_{i} -\boldsymbol{\mathrm{p}}|| + vdW_{i}\} $$

where the unit of measurement for *R*(***p***), *D*(***p***), and *L*(***p***) is expressed in [Å].

### Atomic sampling around the pore

We consider nested sampling spheres centered at ***p*** with radii given by [41]

m4 $$  l_{\alpha}(\boldsymbol{\mathrm{p}}) = D(\boldsymbol{\mathrm{p}}) +\alpha\!\cdot\!\frac{L(\boldsymbol{\mathrm{p}})-D(\boldsymbol{\mathrm{p}})}{K_{\alpha}}\text{ for } \alpha=1,2,..,K_{\alpha}\!\to\!\infty $$

where *K*_*α*_ is the total number of sampling spheres and *α* denotes the index of the sampling sphere (thus, from now on, we will refer to it as the “scaling index”). Accordingly, *l*_*α*_(***p***) indicates the size (or, equivalently, the scale) of the spherical cluster of atoms around ***p*** in [Å]. Then, the number of atoms around ***p*** is given by [41]

m5 $$  N(\boldsymbol{\mathrm{p}},l_{\alpha}(\boldsymbol{\mathrm{p}})) = \sum\limits_{i=1}^{N_{c}} \theta(l_{\alpha}(\boldsymbol{\mathrm{p}}) - ||\boldsymbol{\mathrm{c}}_{i} -\boldsymbol{\mathrm{p}}||) $$

where *𝜃*(⋅) is the Heaviside function.

### Candidate models for describing packing of atoms around the pore

Atom-packing around ***p*** was modeled by employing the GROFIT routine [56] and applying it on Eq. m5. Accordingly, a collection of candidate sigmoid models including re-parametrized algebraic forms [57] of the logistic model [58]

m6 $$  n_{LOG}(\boldsymbol{\mathrm{p}},l_{\alpha}(\boldsymbol{\mathrm{p}})) = A(\boldsymbol{\mathrm{p}}) \!\cdot\! \big \{  1 + \exp\big(\frac{4\!\cdot\!t(\boldsymbol{\mathrm{p}})}{A(\boldsymbol{\mathrm{p}})} \!\cdot\!\big (s(\boldsymbol{\mathrm{p}}) - l_{\alpha}(\boldsymbol{\mathrm{p}})) + 2 \big) \big \}^{-1} $$,

of the Gompertz model [59]

m7 $$  n_{GOM}(\boldsymbol{\mathrm{p}},l_{\alpha}(\boldsymbol{\mathrm{p}})) = A(\boldsymbol{\mathrm{p}}) \!\cdot\! \exp \big (-\exp (\frac{e\!\cdot\!t(\boldsymbol{\mathrm{p}})}{A(\boldsymbol{\mathrm{p}})} \cdot (s(\boldsymbol{\mathrm{p}}) - l_{\alpha}(\boldsymbol{\mathrm{p}})) + 1) \big ) $$

with $e = \exp (1)$, of the modified Gompertz model [56]

m8 $$  \begin{aligned} n_{MGOM}(\boldsymbol{\mathrm{p}},l_{\alpha}(\boldsymbol{\mathrm{p}})) = & A(\boldsymbol{\mathrm{p}})\!\cdot\! \exp \big(-\exp \big(\frac{e\!\cdot\!t(\boldsymbol{\mathrm{p}})}{A(\boldsymbol{\mathrm{p}})} \! \cdot \! (s(\boldsymbol{\mathrm{p}}) - l_{\alpha}(\boldsymbol{\mathrm{p}})) + 1 \big) \big) \\ & + A(\boldsymbol{\mathrm{p}}) \!\cdot \! \exp \big(w(\boldsymbol{\mathrm{p}}) \!\cdot\!(l_{\alpha}(\boldsymbol{\mathrm{p}}) - l_{shift}(\boldsymbol{\mathrm{p}})) \big) \end{aligned} $$

and of the Richards model [44]

m9 $$  \begin{aligned} n_{RIC}&(\boldsymbol{\mathrm{p}},l_{\alpha}(\boldsymbol{\mathrm{p}})) = A(\boldsymbol{\mathrm{p}})\cdot {\big \{ 1 + \tilde{q}(\boldsymbol{\mathrm{p}})\!\cdot\!b(\boldsymbol{\mathrm{p}}) \!\cdot\! \exp \big (-d(\boldsymbol{\mathrm{p}})\!\cdot\!l_{\alpha}(\boldsymbol{\mathrm{p}}) \big ) \big \}}^{-1/\tilde{q}(\boldsymbol{\mathrm{p}})} \\ &\text{ with } b(\boldsymbol{\mathrm{p}})\!= \!\exp \big (1 + \tilde{q}(\boldsymbol{\mathrm{p}}) + d(\boldsymbol{\mathrm{p}})\!\cdot\!s(\boldsymbol{\mathrm{p}}) \big ) \text{ and } d(\boldsymbol{\mathrm{p}}) = \frac{t(\boldsymbol{\mathrm{p}})}{A(\boldsymbol{\mathrm{p}})}\!\cdot\!(1+\tilde{q}(\boldsymbol{\mathrm{p}}))^{1+1/\tilde{q}(\boldsymbol{\mathrm{p}})} \end{aligned} $$

were fitted on *N*(***p***,*l*_*α*_(***p***)) along the *l*_*α*_(***p***)-direction where $\{A(\boldsymbol {\mathrm {p}}),t(\boldsymbol {\mathrm {p}}),s(\boldsymbol {\mathrm {p}}),\tilde {q}(\boldsymbol {\mathrm {p}}),w(\boldsymbol {\mathrm {p}}),l_{shift}(\boldsymbol {\mathrm {p}})\}$ are model parameters.

In order to proceed with model parameters interpretation, we introduced the inflection point of an atom-packing model with

m10 $$  \xi(\boldsymbol{\mathrm{p}})\! = \! \{ l_{\alpha}(\boldsymbol{\mathrm{p}}) | \frac{\partial^{2} n(\boldsymbol{\mathrm{p}},l_{\alpha}(\boldsymbol{\mathrm{p}}))}{\partial l_{\alpha}(\boldsymbol{\mathrm{p}})^{2}}\! =\!0 \} $$

that determines the location along the *l*_*α*_(***p***)-direction where the atomic radial distribution function (RDF), $\frac {\partial n(\boldsymbol {\mathrm {p}},l_{\alpha }(\boldsymbol {\mathrm {p}}))}{\partial l_{\alpha }(\boldsymbol {\mathrm {p}})}$, is maximized (see SI, Fig. S2). Then, parameter *t*(***p***) can be interpreted as the slope of *n*(***p***,*l*_*α*_(***p***)) at *ξ*(***p***) thus accounting for the maximum atom-packing rate (or, equivalently, for the maximum atom-packing density) around ***p***. Note that *t*(***p***) can be written as $t(\boldsymbol {\mathrm {p}}) = \frac {A(\boldsymbol {\mathrm {p}})}{os(\boldsymbol {\mathrm {p}})= o(\boldsymbol {\mathrm {p}})-s(\boldsymbol {\mathrm {p}})}$ with *o*(***p***) determining the location along *l*_*α*_(***p***)-direction where the asymptote domain begins as *A*(***p***) is the asymptote value of the fitted model, i.e., $n(\boldsymbol {\mathrm {p}},l_{\alpha }(\boldsymbol {\mathrm {p}})\!\to \!\infty ) = A(\boldsymbol {\mathrm {p}})$, and with *s*(***p***) determining the location along *l*_*α*_(***p***)-direction where the lag domain ends, i.e., the size of the lag atom-packing domain (in analogy to the “lag phase” parameter appearing in [57]). Parameter $\tilde {q}(\boldsymbol {\mathrm {p}})$ determines the shape of the Richards model curve, as well as the location of the inflection point along *l*_*α*_(***p***)-direction thus serving as a “summary parameter.” Finally, parameters *w*(***p***) and *l*_*shift*_(***p***) of the modified Gompertz model determine the location and the slope, respectively, of a second increase in the modified Gompertz model curve (see [56]). The logistic and the Gompertz models are special cases of the Richards model for $\tilde {q}(\boldsymbol {\mathrm {p}}) = 1$ and $\tilde {q}(\boldsymbol {\mathrm {p}})\!\to \!0$, respectively, as shown in [60]. Additional parameters utilized by the modified Gompertz growth model and the Richards model increase flexibility of fitting experimental sigmoid curves. For a graphical interpretation of Richards-model parameters, see SI, Fig. S2.

### Thermodynamic modeling of the pore

Two types of interactions are relevant for NavChs: (a) an interaction between atomic channel substructures and its surroundings, i.e., a stabilizing interaction, and (b) an interaction of the atomic structure with a pore-permeating species of hydration radius ≈ *R*(***p***) which can “fit” in the pore. A molecular index that can efficiently summarize these interactions is the hydropathic dipole moment [61]. Accordingly, we utilize the cumulative hydropathic dipole field (CHDF) at ***p***, i.e., the hydropathic dipole field effect at ***p*** originating from the spatial arrangement of *N*(***p***,*l*_*α*_(***p***)) atoms around it, given by [41]

m11 $$  \begin{aligned} & \vec{\boldsymbol{h}}(\boldsymbol{\mathrm{p}},l_{\alpha}(\boldsymbol{\mathrm{p}}))\!= \!\sum\limits_{i=1}^{N_{c}}\theta(l_{\alpha}(\boldsymbol{\mathrm{p}}) - ||\boldsymbol{\mathrm{c}}_{i}-\boldsymbol{\mathrm{p}}||)\!\cdot\!HI_{i}^{\chi}\!\cdot\!\vec{\boldsymbol{r}}_{\mathrm{p},i}= \\ & \underbrace{h_{x}(\boldsymbol{\mathrm{p}},l_{\alpha}(\boldsymbol{\mathrm{p}}))\!\cdot\!\hat{\mathbf{x}}+ h_{y}(\boldsymbol{\mathrm{p}},l_{\alpha}(\boldsymbol{\mathrm{p}}))\!\cdot\!\hat{\mathbf{y}}}_{\vec{\boldsymbol{h}}_{xy}(\boldsymbol{\mathrm{p}},l_{\alpha}(\boldsymbol{\mathrm{p}}))}+ \underbrace{h_{z}(\boldsymbol{\mathrm{p}},l_{\alpha}(\boldsymbol{\mathrm{p}}))\!\cdot\!\hat{\mathbf{z}}} _{\vec{\boldsymbol{h}}_{z}(\boldsymbol{\mathrm{p}},l_{\alpha}(\boldsymbol{\mathrm{p}}))} \end{aligned} $$

where $HI_{i}^{\chi } = HI_{i} + \chi _{i}$ is the *i*-th atomic hydropathic index corresponding to the Kapcha-Rossky atomic hydropathic indices [62] (see Suppl. Table S2 in [41]) with additive Gaussian noise $\chi _{i}\!\in \!\mathcal {N}\!(\mu = 0,\sigma = 0.001)$ and $\vec {\boldsymbol {r}}_{i,p}$ is a vector from ***p*** to ***c***_*i*_. Introduction of the weak noise source *χ*_*i*_ practically guarantees that $||\vec {\boldsymbol {h}}(\boldsymbol {\mathrm {p}},l_{\alpha }(\boldsymbol {\mathrm {p}}))||$ is non-zero for every combination of ***p*** and *l*_*α*_(***p***)) while not affecting its spatial behavior. Moreover, the measurement unit of $\vec {\boldsymbol {h}}(\boldsymbol {\mathrm {p}},l_{\alpha }(\boldsymbol {\mathrm {p}}))$ is given in Debye units [kcal⋅Å/mol] [61] with 1 mol representing a cluster of of *N*(***p***,*l*_*α*_(***p***)) atoms.

Due to preservation of structural NavAb symmetries with respect to *P*, the magnitude of the radial field component, $||\vec {\boldsymbol {h}}_{xy}(\boldsymbol {\mathrm {p}},l_{\alpha }(\boldsymbol {\mathrm {p}}))||$, is vanishingly small and, consequently, $||\vec {\boldsymbol {h}}(\boldsymbol {\mathrm {p}},l_{\alpha }(\boldsymbol {\mathrm {p}}))||$ is adequately represented in terms of the magnitude of the axial field component, $||\vec {\boldsymbol {h}}_{z}(\boldsymbol {\mathrm {p}},l_{\alpha }(\boldsymbol {\mathrm {p}}))||$, i.e.,

m12 $$  \begin{aligned} & ||\vec{\boldsymbol{h}}(\boldsymbol{\mathrm{p}},l_{\alpha}(\boldsymbol{\mathrm{p}}))|| = \sqrt{||\vec{\boldsymbol{h}}_{xy}(\boldsymbol{\mathrm{p}},l_{\alpha}(\boldsymbol{\mathrm{p}}))||^{2} + ||\vec{\boldsymbol{h}}_{z}(\boldsymbol{\mathrm{p}},l_{\alpha}(\boldsymbol{\mathrm{p}}))||^{2}}\!\approx\!||\vec{\boldsymbol{h}}_{z}(\boldsymbol{\mathrm{p}},l_{\alpha}(\boldsymbol{\mathrm{p}}))|| \end{aligned} $$

where a computational proof for the validity of (m12) is given in Suppl. of [41]. Consequently, the orientation of the field is described by only two states: an “in” state which is characterized by $\vec {\boldsymbol {h}}_{z}(\boldsymbol {\mathrm {p}},l_{\alpha }(\boldsymbol {\mathrm {p}}))$ pointing towards the IS, i.e., by *h*_*z*_(***p***,*l*_*α*_(***p***)) > 0, and an “out” state which is characterized by $\vec {\boldsymbol {h}}_{z}(\boldsymbol {\mathrm {p}},l_{\alpha }(\boldsymbol {\mathrm {p}}))$ pointing towards the ES, i.e., by *h*_*z*_(***p***,*l*_*α*_(***p***)) < 0 (for a detailed analysis of the CHDF topology emerging from the “in”-vs-“out” interplay across different scales is presented in [41]).

### Testing the non-extensitivity hypothesis for atomic hydropathicity around NavAb’s pore

The hydropathic non-extensitivity hypothesis was tested by investigating how the CHDF behaves relative to the atomic number, *N*(***p***,*l*_*α*_(***p***)), for increasing molecular scale *l*_*α*_(***p***). For that, we scrutinized the scaling behavior of the hydropathic imbalance [48] (or, equivalently, of the inter-atomic hydropathic interaction strength) given by [41]

m13 $$  \begin{aligned} & I(\boldsymbol{\mathrm{p}},l_{\alpha}(\boldsymbol{\mathrm{p}}))=\frac{||\vec{\boldsymbol{h}}_{z}(\boldsymbol{\mathrm{p}},l_{\alpha}(\boldsymbol{\mathrm{p}}))||}{N(\boldsymbol{\mathrm{p}},l_{\alpha}(\boldsymbol{\mathrm{p}}))} \text{[kcal$\cdot$\AA /mol]} \end{aligned} $$

by searching for sets of pore points for which *I*(***p***,*l*_*α*_(***p***)) can be accurately approximated by a power-law function along *l*_*α*_(***p***)-direction. Note that 1 mol in Eq. m13 represents 1 atom. Interpretation of the findings was based on the rationale that a power-law scaling behavior of *I*(***p***,*l*_*α*_(***p***)) implies that the inter-atomic hydropathic interaction laws around ***p*** remain invariant under scale transformations or, equivalently, its range spans up to ≈ *l*_*α*_(***p***) Å. Accordingly, a positive (negative) power-law exponent indicates that the range of hydropathic inter-atomic interactions increases (decreases), i.e., quantifies the rate at which non-local (local) bonds are formed within the atomic structure. The interaction energy associated with bond formation stabilizing the ensemble of *N*(***p***,*l*_*α*_(***p***) atoms around ***p***, i.e., the atom-packing energy (AE), is then be approximated by $||\vec {\boldsymbol {h}}_{z}(\boldsymbol {\mathrm {p}},l_{\alpha }(\boldsymbol {\mathrm {p}}))||/l_{\alpha }(\boldsymbol {\mathrm {p}})$ measured in [kcal/mol].

## Results

Prior knowledge required in order to interpret the findings of this study is retrieved from [41] and summarized in Table 1.

**Table 1:** Geometrical and hydropathic characteristics of the pore’s microenvironment. We present a summary of geometrical and hydropathic characteristics of the selectivity filter, central cavity, and activation gate pore regions in accordance to [31, 41]

Pore region	Location	Geometry	Hydropathicity
Selectivity filter (SF)	p_*z*_ = − 11.7	Narrow	Hydrophilic
Central cavity (CC)	p_*z*_ = 2.8	Wide	Hydrophobic
Activation gate (AG)	p_*z*_ = 22.7	Occluded	Hydropathically-diverse

### Bi-phasic structural organization of the atomic environment around NavAb’s pore

**Fig. 1:**
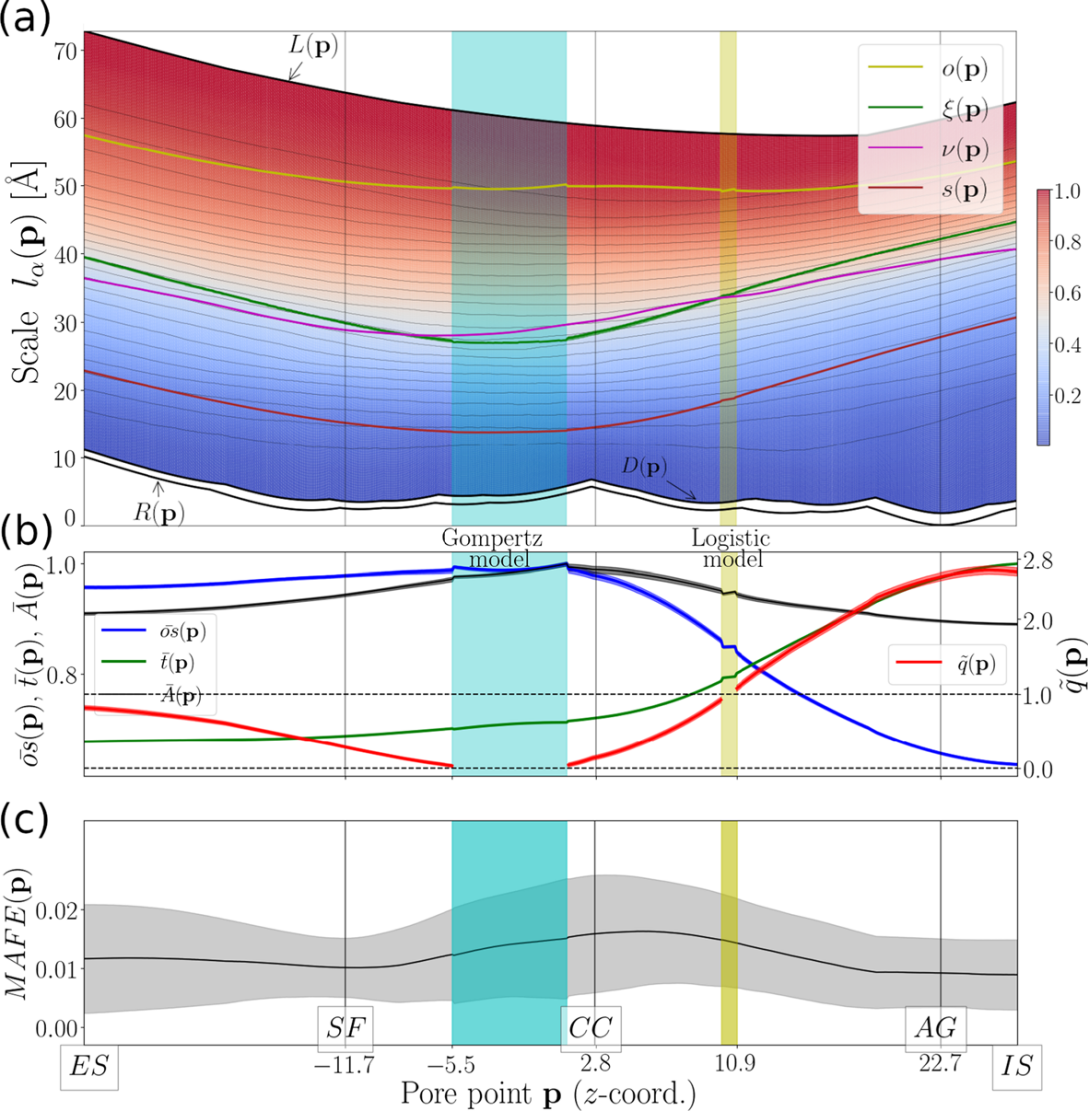
Atom-packing around NavAb’s pore. **a** Contour map of the normalized traces $\bar {N}(\boldsymbol {\mathrm {p}},l_{\alpha }(\boldsymbol {\mathrm {p}}))$ for ***p*** ∈ *Q* and *α* = 1,2,..,*K*_*α*_ = 800 (see SI, Section S2 for construction of *Q*). Black lines *R*(***p***), *D*(***p***), and *L*(***p***) depict pore’s geometrical characteristics. Magenta dashed line *ν*(***p***) serves as a geometrical representation of the geometrical crossover from the PDs to the VSDs (see SI, Section S4). Line *ξ*(***p***) depicts the trace of inflection points, and lines *s*(***p***) and *o*(***p***) depict the ending and beginning of the lag and asymptote domain, respectively. **b** Traces of normalized model parameters $\bar {A}(\boldsymbol {\mathrm {p}})$, $\bar {t}(\boldsymbol {\mathrm {p}})$, $\bar {os}(\boldsymbol {\mathrm {p}})$ and of summary model parameter $\tilde {q}(\boldsymbol {\mathrm {p}})$ for ***p*** ∈ *Q*. **c** Mean absolute fitting error (MAFE) of the mathematical atom-packing model approximation on $\bar {N}(\boldsymbol {\mathrm {p}},l_{\alpha }(\boldsymbol {\mathrm {p}}))$ for ***p*** ∈ *Q*. Vertical lines roughly indicate the pore regions of the selectivity filter (SF), of the central cavity (CC) and of the activation gate (AG) (see Fig. 2 in [41]). All normalizations were performed with respect to the maximum values of the corresponding traces. ES stands for extracellular side. IS stands for intracellular side. Shaded area around model-parameter traces indicate confidence intervals

In this section, we present a mathematical model for describing atom-packing around NavAb’s pore. This modeling approach is based on the ansatz that the scaling behavior of the atomic number (see Eq. m5 and Fig. 1a for an illustration) corresponds to a sigmoid function with inflection points correlating with the conserved NavCh architectural motif that dictates the structural and functional separation of the pore domains (PDs) from the voltage-sensing domains (VSDs). The validity of this hypothesis was tested by executing the GROFIT [56] algorithm on the normalized atomic-number traces (see caption of Fig. 1). In computational practice, at every pore point, we calculated the corresponding atomic-number trace and fed it into the GROFIT routine. The mathematical model that best fitted normalized atomic-number traces along *l*_*α*_(***p***)-direction was then selected by GROFIT from a pool of candidate atom-packing models (see “Methods,” Section 4) based on minimization of an Akaike information criterion while confidence intervals of model parameters were estimated by using a non-parametric bootstrap method (see [56] for algorithmic details).

Our modeling procedures demonstrate that atom-packing around NavAb’s pore can be accurately described by the Richards model (see Eq. m9) and special cases of it, such as the logistic model (see Eq. m6) and the Gompertz model (see Eq. m7). This becomes obvious if we focus on the trace of the summary parameter, $\tilde {q}(\boldsymbol {\mathrm {p}})$, approaching unity and zero for $\{\mathrm {\mathrm {p}}_{z}\!\to \!-5.5^{-},\mathrm {\mathrm {p}}_{z}\!\to \!1.2^{+}\}$ and for $\{\mathrm {\mathrm {p}}_{z}\!\to \!10.0^{-},\mathrm {\mathrm {p}}_{z}\!\to \!10.9^{+}\}$, thus indicating a smooth transition from the Richards model pore regions towards the Gompertz and logistic model pore regions, respectively. Note that discontinuities arising at the boundaries [5.5,1.2] and [10.0,10.9] are due to numerical artifacts introduced by the GROFIT solver (Fig. 1b). The Gompertz and logistic model pore regions are located on the left and right of the hydrophobic central cavity (CC), thus capturing the transition from the pore center towards the SF and the AG (see Table 1 and Fig. 1b). The accuracy of the Richards model modeling approximation is demonstrated not only by the smallness of absolute fitting error but also by narrow confidence intervals of model parameters (see Fig. 1c).

A direct consequence of the applicability of the Richards model is that the atomic environment around the pore can be partitioned into three consecutive geometrical domains, namely, a lag domain (for *l*_*α*_(***p***) ≤ *s*(***p***)), an inflection domain (for *s*(***p***) < *l*_*α*_(***p***) ≤ *o*(***p***)), and an asymptote domain (for *l*_*α*_(***p***) > *o*(***p***)) (Fig. 1a and SI, Fig. S2). Approximately 95*%* of the lag domain is structurally composed of pore-lining components belonging to the S5–S6 helices while the rest (5*%*) is drawn from the S4–S5 linkers. The inflection domain accounts for a structurally diverse environment incorporating parts from both the S5–S6 helices and the S1–S4 voltage-sensing segments. Lastly, the asymptote domain is solely populated by VSD-forming residues with the majority (55*%*) of them belonging to the far-distant (from the pore axis) S1 segments.

Size variations of the inflection domain described by *os*(***p***) parameter (see “Methods”) indicate how atom-packing density is changing along the pore axis. In particular, within the Gompertz model pore region, the size of the inflection domain maximizes while the atom-packing rate, *t*(***p***) (see “Methods”), is downregulated so that a weakly dense atomic environment is formed at the extracellular side (ES) of the CC (Fig. 1a, and b). In contrast, deviating from the Gompertz model pore region towards the intracellular side (IS), *os*(***p***) decreases while *t*(***p***) increases so that a strongly dense atomic environment is formed around the AG implementing pore occlusion (Fig. 1a and b). This qualitative change in atom-packing conditions is captured by the logistic model pore region as the summary parameter, $\tilde {q}(\boldsymbol {\mathrm {p}})$ (see “Methods”), becomes larger (smaller) than one for p_*z*_ > 10.9 (p_*z*_ < 10.0) (Fig. 1b). Deviating from the Gompertz model pore region towards the extracellular side (ES), both *os*(***p***) and *t*(***p***) exhibit a weakly decreasing tendency indicative of a structural tendency for uniform atom-packing conditions.

Biophysical importance of the inflection mechanism becomes evident if we consider how our observations correlate with the spatial organization of the PDs-forming structural elements relatively to the VSDs-forming structural elements. For that, we approximated the scales at which the spatial transition, i.e., the geometrical crossover, from the PDs to the VSDs takes place (see SI, Section S4). We found that the traces of inflection points, *ξ*(***p***)) (see Eq. m10), and of the PDs-VSDs geometrical crossover follow closely each other with their mutual distance on the contour map of Fig. 1a given by 1.82 ± 0.94 Å and with Spearman correlation scores of 0.92 and 0.99 for p_*z*_ > 0 and p_*z*_ ≤ 0, respectively. Inflection points are thus indicators of the distance from the pore axis at which the structural separation of the PDs from the VSDs takes place. Accordingly, we may consider only two structural phases, namely, pre- and post-inflection phases realized for *l*_*α*_(***p***) ≤ *ξ*(***p***) and *l*_*α*_(***p***) > *ξ*(***p***), respectively, reflecting the well-conserved PDs-VSDs dichotomy. Maximization of atom-packing density for *l*_*α*_(***p***) ≈ *ξ*(***p***) can result in strengthening of inter-atomic interactions which, in turn, favors coupling and relative stability of the PDs and VSDs atomic subsystems.

### Non-extensitivity of atomic hydropathicity around NavAb’s pore

What do observations of the previous section imply for the nature of underlying inter-atomic stabilizing interactions? To answer this question, let us recall here that stability of atom-packing around protein cores is largely determined by hydropathic effects [63]. Following this rationale, we examined the scaling behavior of the hydropathic inter-atomic interactions strength (HIIS) (see Eq. m13) with respect to the pre- and post-inflection geometrical intervals which essentially test the validity of the non-extensitivity hypothesis.

**Fig. 2:**
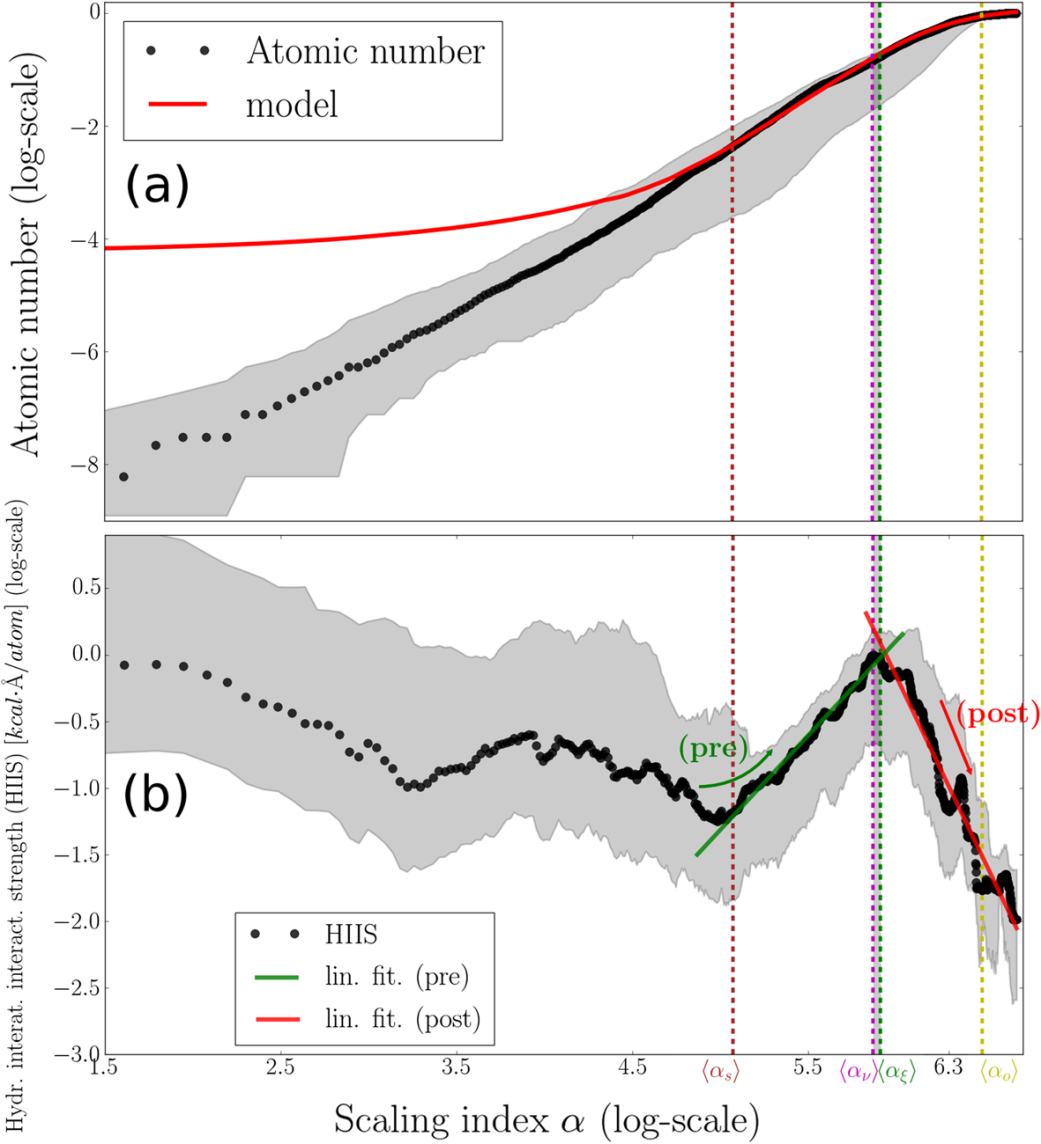
Non-extensitivity of atomic hydropathicity around the NavAb’s pore. **a** Trace of a statistical representation of the normalized atomic number, $\langle \bar {N}(\boldsymbol {\mathrm {p}},l_{\alpha }(\boldsymbol {\mathrm {p}})) \rangle _{\alpha }$, and of its best-fitted model, 〈*n*(***p***,*l*_*α*_(***p***))〉_*α*_, are plotted in log-scale. 〈*n*(***p***,*l*_*α*_(***p***))〉_*α*_ corresponds to the Richards model. **b** Trace of a statistical representation of the hydropathic imbalance magnitude, 〈*I*(***p***,*l*_*α*_(***p***))〉_*α*_, is plotted with its linear fittings for 〈*α*_*s*_〉 < *α* ≤ 〈*α*_*ξ*_〉 and *α* > 〈*α*_*ξ*_〉, respectively, in log-scale. Up- and downregulation tendencies were quantified in terms of the linear fittings $\gamma \!\cdot \!\log [\alpha ]+\upbeta $ with *γ*_*pre*_ = 1.41 and β_*pre*_ = − 8.4, *γ*_*post*_ = − 2.76 and β_*post*_ = 16.43. The corresponding Pearson coefficients are *PC*_*pre*_ = 0.99 and *PC*_*post*_ = − 0.97, respectively, indicating the “goodness” of the regulation. Error bars in **a** and **b** represent 95*%* interval values. For clarity, the size of error bars in **b** is reduced by a factor of 0.5. Calculation of $\langle \bar {N}(\boldsymbol {\mathrm {p}},l_{\alpha }(\boldsymbol {\mathrm {p}})) \rangle _{\alpha }$, 〈*n*(***p***,*l*_*α*_(***p***))〉_*α*_, 〈*I*(***p***,*l*_*α*_(***p***))〉_*α*_, 〈*α*_*s*_〉, 〈*α*_*ξ*_〉, 〈*α*_*ν*_〉, and 〈*α*_*o*_〉 was performed according to the statistical scheme presented in SI, Section S5. All normalizations were performed with respect to the maximum values of the corresponding traces

Coarse-grained computational evidence for the validity of the non-extensitivity hypothesis is presented in Fig. 2. We show that maximization of HIIS is expected to take place within the narrow interval *ν*(***p***) < *l*_*α*_(***p***) ≤ *ξ*(***p***), thus identifying the PDs-VSDs geometrical crossover as a strong-interactions intra-channel site. Moreover, the spatial transition from the lag domain to the inflection domain is associated with a local minimization of HIIS followed by its upregulation and, eventually, maximization up to *l*_*α*_(***p***) ≈ *ξ*(***p***) (Fig. 2). Below this regime, HIIS is downregulated toward its global minimization at the end of the asymptote domain (see caption of Fig. 2). These observations indicate a “tuning” of HIIS scaling behavior with respect to the structural classification scheme presented in Section I. From a stability viewpoint, the main advantage of this configuration is that the HIIS between the PDs and the VSDs is maximized within a narrow window around *ν*(***p***), thus optimizing the PDs-VSDs coupling. Within the lag domain, our modeling procedures appear to be much less efficient in describing cumulative atomic properties as indicated by the increase in deviations between fitted models and empirical NavAb data (Fig. 2a and b). This modeling impotence indicates that within lag-domain scales, HIIS scaling analysis suffers from small-sized sampling which, in turn, suggests the existence of a cutoff scale *l*_*α*_(***p***) ≈ *s*(***p***) above which cumulative hydropathic effects become an important driving force of atom-packing. Equivalently, within the lag domain, HIIS exhibits non-trivial short-range dependencies which might originate from water structuring effects at the wall surfaces prevailing within scales smaller than 15 Å [40]. However, given that HIIS exhibits a pseudo-symmetric spatial profile with respect to the center of the pore coinciding with the CC center, hydropathic effects originating from scales smaller than *s*(***p***) have a major contribution to pore gating behavior, and hence to ion de/re-hydration dynamics as demonstrated in [41].

**Fig. 3:**
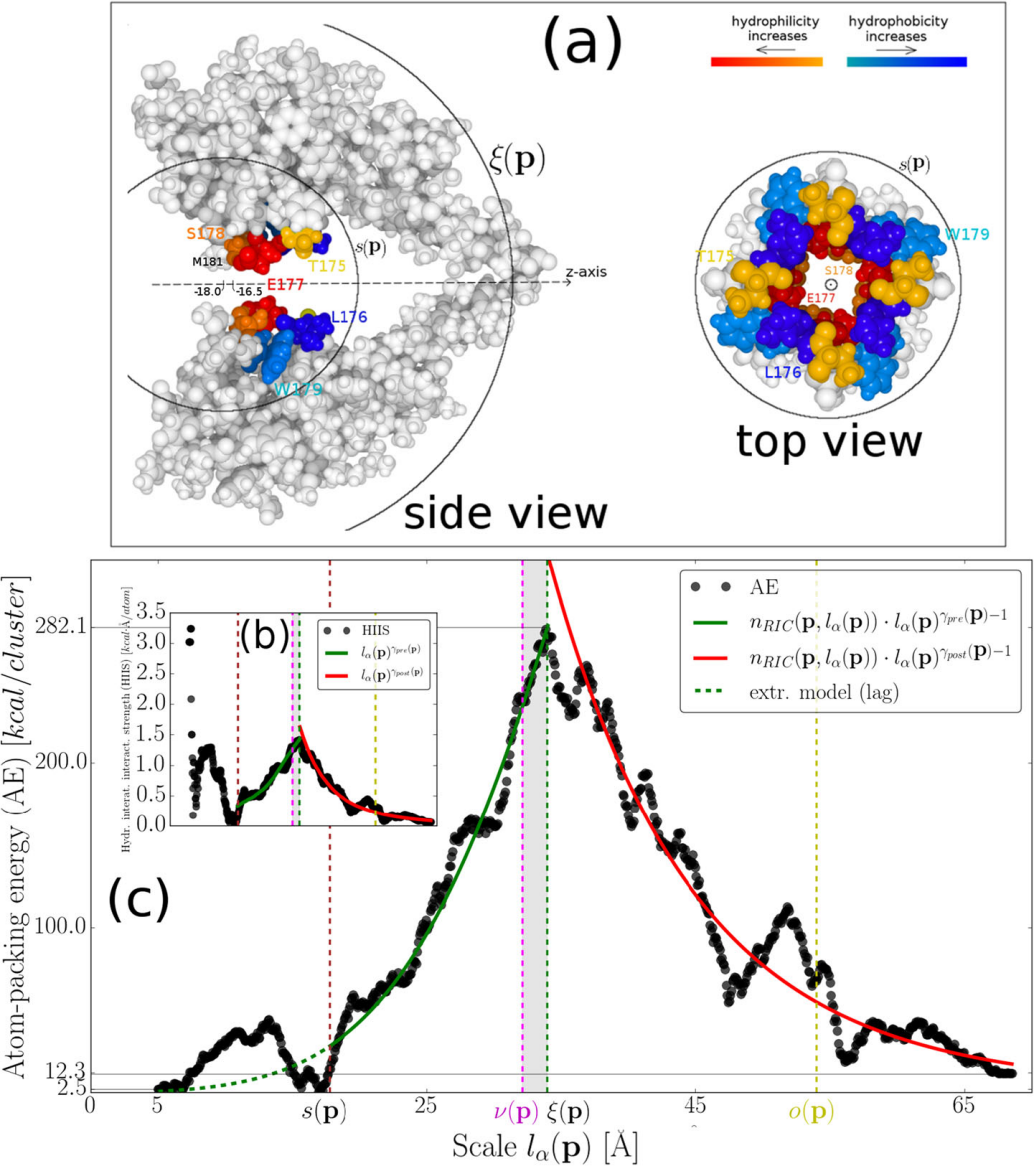
Non-extensive modeling of inter-atomic hydropathic interactions around NavAb’s pore. **a** Cartoon illustration of two opposite-facing PD structural units. The radii *s*(***p***) and *ξ*(***p***) centered at the middle of p_*z*_ ∈ [− 18.0,− 16.5] roughly account for the scales at which the lag domain and the pre-inflection domains end, i.e., for the sizes of the lag and pre-inflection domain, respectively. Residues *T*175, *L*176, *E*177, *S*178, and *W*179 forming the selectivity filter are colored according to their hydropathic score based on the Kapcha-Rossky scale. p_*z*_ ∈ [− 18.0,− 16.5] is directly neighbored by atomic components forming the *M*181 and *S*178 residue side chains. **b** Trace of the hydropathic inter-atomic interaction strength (HIIS), *I*(***p***,*l*_*α*_(***p***)), for a randomly chosen pore point from the pore region p_*z*_ ∈ [− 18.0,− 16.5]. The best-fitting pre- and post-inflection power-law approximations of HIIS are also plotted with their mean absolute relative fitting errors being 0.089 ± 0.004 and 0.21 ± 0.008, respectively. **c** Trace of the corresponding atom-packing energy (AE), $||\vec {\boldsymbol {h}}_{z}(\boldsymbol {\mathrm {p}},l_{\alpha }(\boldsymbol {\mathrm {p}}))||/l_{\alpha }(\boldsymbol {\mathrm {p}})$. Pre- and post-inflection modeling approximations of AE are also plotted with their mean absolute relative modeling errors being 0.096 ± 0.005 and 0.21 ± 0.009, respectively. Model extrapolation toward *l*_*α*_(***p***) ≤ *s*(***p***) results in a mean absolute relative fitting error of 1.77 ± 0.47. *ν*(***p***) and *o*(***p***) account for the scales at which the PDs-VSDs geometrical crossover occurs and the asymptote domain begins, respectively

Fine-grained analysis of the scaling behavior of HIIS revealed a clear enhancement of its scale-invariant character at the ES of the highly conserved SF (see SI, Fig. S4, and Fig. 3a and b). Specifically, out of all possible atom-packing paths initiated along the pore axis, scale invariance occurs only for − 18.0 ≤ p_*z*_ ≤ − 16.5 corresponding to a pore region which is directly neighbored by the hydrophobic *M*181 residue side chain belonging to the P2-helix, as well as, by the hydrophilic *S*178 residue side chains belonging to the SF (see Fig. 3a and [41] for direct-neighboring residues to corresponding ***p***). From the perspective of a pore-permeating ion species, this atom-packing configuration implies a large-amplitude, short-range interaction exerted upon incoming ions from NavAb’s walls as indicated by large values of HIIS for *l*_*α*_(***p***) ≤ *s*(***p***) (Fig. 3b). Indeed, the SF residue sequence *T*175:*W*179 is part of this atomic configuration as the sampling radius *s*(***p***) encloses its structure as a whole (Fig. 3a). Below the lag scales, it is straightforward to approximate the amplitude of the CHDF (see (m12)) for − 18.0 ≤ p_*z*_ ≤ − 16.5 with the following power-law scheme

r1 $$  \begin{aligned} & ||\vec{\boldsymbol{h}}_{z}(\boldsymbol{\mathrm{p}},l_{\alpha}(\boldsymbol{\mathrm{p}}))|| = \begin{cases} -h_{z}(\boldsymbol{\mathrm{p}},l_{\alpha}(\boldsymbol{\mathrm{p}})) \sim n_{RIC}(\boldsymbol{\mathrm{p}},l_{\alpha}(\boldsymbol{\mathrm{p}}))\cdot l_{\alpha}(\boldsymbol{\mathrm{p}})^{\gamma_{pre}(\boldsymbol{\mathrm{p}})} \text{ for } s(\boldsymbol{\mathrm{p}})\!<\!l_{\alpha}(\boldsymbol{\mathrm{p}})\!\leq\!\xi(\boldsymbol{\mathrm{p}}) \\ -h_{z}(\boldsymbol{\mathrm{p}},l_{\alpha}(\boldsymbol{\mathrm{p}})) \sim n_{RIC}(\boldsymbol{\mathrm{p}},l_{\alpha}(\boldsymbol{\mathrm{p}}))\cdot l_{\alpha}(\boldsymbol{\mathrm{p}})^{\gamma_{post}(\boldsymbol{\mathrm{p}})} \text{ for } l_{\alpha}(\boldsymbol{\mathrm{p}})\!>\!\xi(\boldsymbol{\mathrm{p}}) \end{cases} \end{aligned} $$

with *γ*_*pre*_(***p***) = 2.27 ± 0.1 and *γ*_*post*_(***p***) = − 4.36 ± 0.09 being the pre- and post-inflection scaling exponents (see Fig. 3b, and SI, Fig. S3), and *N*(***p***,*l*_*α*_(***p***)) replaced with its best-fitted model, namely, the Richards-model parameters for $\{A(\boldsymbol {\mathrm {p}})\!\approx \!1.05,t(\boldsymbol {\mathrm {p}})\!\approx \! 0.2,s(\boldsymbol {\mathrm {p}})\!\approx \!18.02,\tilde {q}(\boldsymbol {\mathrm {p}})\!\approx \!0.54\}$. Topological invariance of $\vec {\boldsymbol {h}}_{z}(\boldsymbol {\mathrm {p}},l_{\alpha }(\boldsymbol {\mathrm {p}}))$ (as indicated by the negative sign of *h*_*z*_(***p***,*l*_*α*_(***p***)) at the rhs of (r1)) allows for an interpretation of the observed scaling laws. The atom-packing energy (AE) associated with this CHDF configuration is retrieved by dividing (r1) with *l*_*α*_(***p***) (see Fig. 3c and its caption for the accuracy of the approximation). This estimation scheme predicts a PDs-VSDs coupling energy of $\sim \!282.1$ kcal as AE maximizes within the narrow window [*ν*(***p***),*ξ*(***p***)] (Fig. 3c). Microscopic building blocks of the CHDF are dipole-dipole interactions (e.g., hydrogen bonds) formed between neighboring atoms. Each of these short-range interactions can be assigned with a dipole moment. The physical meaning attributed then to Eq. r1 is that the mean-field amplitude and orientation of these dipole moments remain invariant for increasing system size. Consequently, in the pre-inflection phase, a long-range stabilizing effect emerges reflecting a non-random alignment of dipoles with respect to − 18.0 ≤ p_*z*_ ≤ − 16.5 across different NavAb scales. Equivalently, we may think of this stabilizing mechanism as an interactions network [31] expanding throughout the PDs with a rate that is quantified in terms of *γ*_*pre*_(***p***). The *E*177 side chains were previously identified as an interactions network center along the pore; they form bonds with their direct atomic environment which extend throughout the P- and P2-helices and, most likely, throughout the PDs as a whole (see Suppl., Fig.10 in [31]). Our findings closely match this anticipation although they predict a network-initiator role for a pore-lining cluster of *M*181 and *S*178 atomic components found at the ES of the SF (Fig. 3a). In the post-inflection phase, spatial dynamics is reversed as the network contracts with a rate of |*γ*_*post*_(***p***)| indicative of short-rangeness rebound and energy dissipation due to bond breaking. Notably, |*γ*_*post*_(***p***)| is almost double the size as *γ*_*pre*_(***p***) indicating that breaking of long-range bonds in favor of short-range ones is a “faster” process than creating them. This fast rebound of bonding-locality might provide energy surplus for stabilization of the NavAb within a lipid environment as the atom-packing energy of the largest atomic cluster (≈ 12.3 kcal) is larger than that of the initial atomic cluster (≈ 2.5 kcal) (Fig. 3c).

In summary, the main advantage behind the CHDF configuration described by (r1) is that the ratio $\frac {||\vec {\boldsymbol {h}}_{z}(\boldsymbol {\mathrm {p}},l_{\alpha }(\boldsymbol {\mathrm {p}}))||}{N(\boldsymbol {\mathrm {p}},l_{\alpha }(\boldsymbol {\mathrm {p}}))}$ does not depend anymore on microscopic details of the atomic environment which implies that HIIS involved in stable functioning of the SF exhibits robustness to structural channel modifications such as point mutations. For example, let us consider the case of an amino acid substitution occurring at distance *l*_*α*_(***p***) ≈ *ξ*(***p***) from the SF. This mutation event can lead to a re-distribution of atoms around the SF which can be expressed as a perturbation of the form *N*(***p***,*l*_*α*_(***p***)) → *N*(***p***,*l*_*α*_(***p***)) + *𝜖*(***p***,*l*_*α*_(***p***)) where *𝜖*(***p***,*l*_*α*_(***p***)) is a perturbation additive source quantifying the size of mutation-induced change in the atomic number. In turn, this will result in a perturbation of the CHDF magnitude of the form $||\vec {\boldsymbol {h}}_{z}(\boldsymbol {\mathrm {p}},l_{\alpha }(\boldsymbol {\mathrm {p}}))||\to ||\vec {\boldsymbol {h}}_{z}(\boldsymbol {\mathrm {p}},l_{\alpha }(\boldsymbol {\mathrm {p}}))|| + \zeta (\boldsymbol {\mathrm {p}},l_{\alpha }(\boldsymbol {\mathrm {p}}))$ that, due to (r1), is regulated by $||\vec {\boldsymbol {h}}_{z}(\boldsymbol {\mathrm {p}},l_{\alpha }(\boldsymbol {\mathrm {p}}))|| + \zeta (\boldsymbol {\mathrm {p}},l_{\alpha }(\boldsymbol {\mathrm {p}})) \sim l_{\alpha }(\boldsymbol {\mathrm {p}})^{\gamma (\boldsymbol {\mathrm {p}})}\cdot \{N(\boldsymbol {\mathrm {p}},l_{\alpha }(\boldsymbol {\mathrm {p}}))+\epsilon (\boldsymbol {\mathrm {p}},l_{\alpha }(\boldsymbol {\mathrm {p}}))\}$ so that the HIIS law remains intact (note that *ζ*(***p***,*l*_*α*_(***p***)) is a perturbation additive source quantifying the size of mutation-induced change in the CHDF magnitude).

## Discussion

An algebraic relationship between $\tilde {q}(\boldsymbol {\mathrm {p}})$ and the non-extensive entropic index, *q*(***p***) [36], is established with $\tilde {q}(\boldsymbol {\mathrm {p}}) = 1-q(\boldsymbol {\mathrm {p}})$ [45]. Hence, Eq. r1 illustrates how the CHDF emerges from the spatial arrangement of atoms around the pore and establishes a direct link with the non-extensive entropic *q*(***p***)-index. This is the main finding of this study and highlights the fact that hydropathic forces are of entropic nature; observations presented in Fig. 3 can be understood as an entropic re-configuration of the atomic environment, i.e., phase transition of the crystal structure (thought of as a glass system [49]), due to re-organization in the interactions range. The critical scale *ξ*(***p***) determines then the maximum bond length of the underlying interactions. Symmetry breaking associated with the critical transition is shown in Fig. 2 of [41] where pseudo-symmetric topological pairs formed within the pre-inflection phase gradually dissolve below *ξ*(***p***).

The long-rangeness of the HIIS around the SF might be crucial for the stable functioning of the biological machinery interacting with selected ions. In particular, ions entering the NavAb’s SF pore region from the ES are expected to strongly interact with pore-lining components at subsequent interactions sites [31] corresponding to negative minima of the energy landscape [41]. These interactions will result in their partial de-hydration and, consequently, heat exchange with the surrounding atomic environment. To counteract for this local increase in pore pressure due to water removal, a non-local interactions network provides peripheral structural support to the biological machinery implementing de-hydration. Indeed, long-range interactions are thought to play a role in selectivity by indirectly influencing the stability of SF’s de-hydration binding sites [64, 65].

Mutations affecting the SF of NavChs can have dramatic effects on channel function (e.g., a single amino acid substitution can alter ion selectivity [66]) and pose a high risk for detrimental destabilizations [67]. The long-rangeness of the HIIS provides a “shield” against potentially destabilizing mutations as HIIS robustness against structural modifications is enhanced. Accordingly, mutation-induced AE perturbations originating from mutations affecting the PDs are expected to be damped-out toward the lag domain according to $\zeta (\boldsymbol {\mathrm {p}},l_{\alpha }(\boldsymbol {\mathrm {p}})) \sim (\xi (\boldsymbol {\mathrm {p}})-l_{\alpha }(\boldsymbol {\mathrm {p}}))^{-\gamma _{pre}(\boldsymbol {\mathrm {p}})}\cdot \epsilon (\boldsymbol {\mathrm {p}},l_{\alpha }(\boldsymbol {\mathrm {p}}))$, thus not affecting pore-lining SF components. Similarly, mutation-induced AE perturbations originating from mutations affecting the VSDs are expected to be damped-out toward the asymptote domain according to $\zeta (\boldsymbol {\mathrm {p}},l_{\alpha }(\boldsymbol {\mathrm {p}})) \sim (l_{\alpha }(\boldsymbol {\mathrm {p}}) - \xi (\boldsymbol {\mathrm {p}}))^{\gamma _{post}(\boldsymbol {\mathrm {p}})}\cdot \epsilon (\boldsymbol {\mathrm {p}},l_{\alpha }(\boldsymbol {\mathrm {p}}))$, thus not affecting channel’s energetic contact with neighboring molecules (e.g., membrane lipids). What is at risk, however, here? Mutation-induced AE perturbations originating either from the PDs or the VSDs can be amplified toward an inflection point which might jeopardize the relative stability of the PDs and VSDs coupled subsystems. Conformational flexibility of the SF and its surroundings (observed via molecular dynamics simulations in [68]) is thus necessary so that potential AE surpluses would be absorbed.

## Conclusions

Critical points correspond to extrema in some property (or properties) with respect to which the protein system under scrutiny has been optimized via long-range and highly cooperative interactions such as these described under the umbrella-term “hydropathic effects” [49, 50]. In this study, we provided with computational evidence for the validity of the atomic hydropathicity non-extensitivity hypothesis for the pre-open NavAb, which directly implies the existence of critical points associated with the stability of the closed-pore NavAb state. Specifically, we showed that the atomic environment around the pore of the pre-open NavAb is organized with respect to well-conserved molecular locations associated with the modular PDs-VSDs architectural motif and with the SF atomic structure. A direct consequence of this is that hydropathic interactions strength exhibits a bi-phasic, scale-invariant behavior that can be modeled in terms of the Richards model parametric space $\{A(\boldsymbol {\mathrm {p}}),t(\boldsymbol {\mathrm {p}}),s(\boldsymbol {\mathrm {p}}),\tilde {q}(\boldsymbol {\mathrm {p}})=1-q(\boldsymbol {\mathrm {p}})\}$ and critical exponents {*γ*_*pre*_(***p***),*γ*_*post*_(***p***)}. Hence, our modeling procedures can provide a reduction of the spatial complexity of the NavCh atomic system under scrutiny under meaningful biological and evolutionary constraints. This is important for channel protein design, mutation-response analysis, and pharmacological control where spatial complexity of the atomic environment poses major challenges (e.g., see [69], [70], and [71], respectively).

## Electronic supplementary material

(PDF 1.90 MB)
