# Correction to: Non-extensitivity and criticality of atomic hydropathicity around a voltage-gated sodium channel’s pore: a modeling study

**DOI:** 10.1007/s10867-022-09616-w

**Published:** 2022-11-12

**Authors:** Markos N. Xenakis, Dimos Kapetis, Yang Yang, Jordi Heijman, Stephen G. Waxman, Giuseppe Lauria, Catharina G. Faber, Hubert J. Smeets, Patrick J. Lindsey, Ronald L. Westra

**Affiliations:** 1 Department of Toxicogenomics, Section Clinical Genomics, Maastricht University, PO Box 616, 6200 MD, Maastricht, The Netherlands; 2 Research School for Mental Health and Neuroscience (MHeNS), Maastricht University, PO Box 616, 6200 MD, Maastricht, The Netherlands; 3 Neuroalgology Unit, Fondazione IRCCS Istituto Neurologico “Carlo Besta”, Via Celoria 11, 20133, Milan, Italy; 4 Department of Medicinal Chemistry and Molecular Pharmacology, Purdue University College of Pharmacy, IN 47907 West Lafayette, USA; 5 Purdue Institute for Integrative Neuroscience, IN 47907 West Lafayette, USA; 6 Department of Cardiology, CARIM School for Cardiovascular Diseases, Maastricht University, PO Box 616, 6200 MD, Maastricht, The Netherlands; 7 Department of Neurology and Center for Neuroscience and Regeneration Research, Yale University School of Medicine, New Haven, CT 06510 USA; 8 Rehabilitation Research Center, Veterans Affairs Connecticut Healthcare System, West Haven, CT 06516 USA; 9 Department of Biomedical and Clinical Sciences, “Luigi Sacco”, University of Milan, Via G.B. Grassi 74, 20157 Milan, Italy; 10 Department of Neurology, Maastricht University Medical Center, PO Box 5800, 6202 AZ, Maastricht, The Netherlands; 11 Research School for Oncology and Developmental Biology (GROW), Maastricht University, PO Box 616, 6200 MD, Maastricht, The Netherlands; 12 Department of Data Science and Knowledge Engineering, Maastricht University, PO Box 616, 6200 MD, Maastricht, The Netherlands

**Correction to: Journal of Biological Physics (2021) 47(1):61–77** https://doi.org/10.1007/s10867-021-09565-w

In this article, the first name of the first author was misspelled: The correct name is Markos N. Xenakis and not Makros N. Xenakis.

The original article has been corrected.

