# Supplementary material for: Non-extensitivity and criticality of atomic hydropathicity around a voltage-gated sodium channel’s pore: a modeling study
Source: J Biol Phys. 2021 Mar 18;47(1):61–77. doi: 10.1007/s10867-021-09565-w (PMC7981368; doi:10.1007/s10867-021-09565-w)
Supplement: Supplementary file 1 — (PDF 1.90 MB) [file 10867_2021_9565_MOESM1_ESM.pdf]

Non-extensitivity and criticality of atomic hydropathicity around a voltage-gated sodium channel's pore; a modeling study

Makros N. Xenakis[a,b,*], Dimos Kapetis[c], Yang Yang[d,e], Jordi Heijman[f], Stephen G. Waxman[g,h], Giuseppe Lauria[c,i], Catharina G. Faber[j], Hubert J. Smeets[a,b], Patrick J. Lindsey[a,k], Ronald L. Westra[l]

[a]*Department of Toxicogenomics, Section Clinical Genomics, Maastricht University, PO Box 616, 6200 MD Maastricht, the Netherlands*
[b]*Research School for Mental Health and Neuroscience (MHeNS), Maastricht University, PO Box 616, 6200 MD Maastricht, The Netherlands*
[c]*Neuroalgology Unit, Fondazione IRCCS Istituto Neurologico "Carlo Besta", via Celoria 11, 20133 Milan, Italy*
[d]*Department of Medicinal Chemistry and Molecular Pharmacology, Purdue University College of Pharmacy, West Lafayette, IN, 47907, USA*
[e]*Purdue Institute for Integrative Neuroscience, West Lafayette, IN 47907, USA*
[f]*Department of Cardiology, CARIM School for Cardiovascular Diseases, Maastricht University, PO Box 616, 6200 MD Maastricht, The Netherlands*
[g]*Department of Neurology and Center for Neuroscience and Regeneration Research, Yale University School of Medicine, New Haven, CT 06510, USA.*
[h]*Rehabilitation Research Center, Veterans Affairs Connecticut Healthcare System, West Haven, CT 06516, USA.*
[i]*Department of Biomedical and Clinical Sciences "Luigi Sacco", University of Milan, via G.B. Grassi 74, 20157 Milan, Italy*
[j]*Department of Neurology, Maastricht University Medical Center, PO Box 5800, 6202 AZ Maastricht, The Netherlands*
[k]*Research School for Oncology and Developmental Biology (GROW), Maastricht University, PO Box 616, 6200 MD Maastricht, the Netherlands*
[l]*Department of Data Science and Knowledge Engineering, Maastricht University, PO Box 616, 6200 MD Maastricht, the Netherlands*

---

*Correspondence and requests for materials should be addressed to M.N.X. E-mail: mrksxenakis@gmail.com

Supplementary Information

S1. The pre-open I217C NavAb structural model

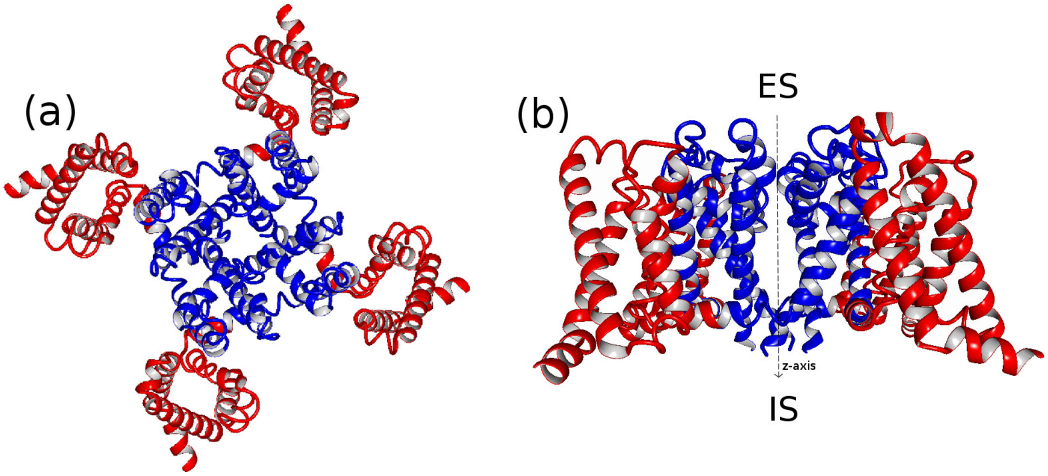

Figure S1. Cartoon illustration of the pre-open I217C NavAb channel. (a), top-view from the extracellular side (ES) towards the intracellular side (IS). (b), side-view. Helices forming the pore domains are illustrated in blue color (residue sequence: $M130$:$M221$). Helices forming the voltage-sensor domains are illustrated in red color (residue sequence: $M001$:$G129$).

S2. Discretization of the principal pore axis

Following [1] we introduced the line grid $Q = \{\mathbf{p}_1, \mathbf{p}_1{+}\Delta\mathbf{p}, .., \mathbf{p}_{N_\mathrm{p}}{-}\Delta\mathbf{p}, \mathbf{p}_{N_\mathrm{p}}\} \subset P$, where N_p is the total number of grid pore points, $||\Delta\mathbf{p}||$ is the sampling distance between two consecutive pore points and $\mathbf{p}_1 = (0, 0, \mathrm{p}_{z,1})$, $\mathbf{p}_{N_\mathrm{p}} = (0, 0, \mathrm{p}_{z,N_\mathrm{p}})$ are boundary pore points. Q is constructed by setting $\mathrm{p}_{z,1} = round(\underset{i=1,2,..,N_c}{min}(c_{z,i}), 1) = -27.1$ and $\mathrm{p}_{z,N_\mathrm{p}} = round(\underset{i=1,2,..,N_c}{max}(c_{z,i}), 1) = 26.8$ with $round(x \in \mathbb{R}, n \in \mathbb{Z}^+)$ returning the value of x rounded upto the n-th decimal digit and by setting $N_\mathrm{p} = 540$ so that $||\Delta\mathbf{p}|| = 0.1$ Å.

S3. Graphical interpretation of atom-packing parameters

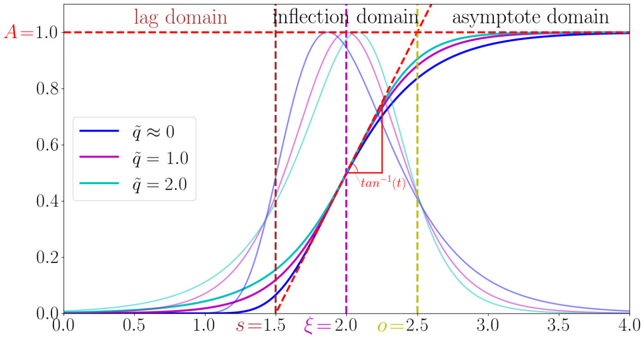

Figure S2. Richards model exemplary traces. The Richards model curve is illustrated for $A = 1$, $t = 1$, $s = 1.5$ and for $\tilde{q} \approx 0.0$, $\tilde{q} = 1.0$ and $\tilde{q} = 2.0$, where A determines the model's asymptotic behavior, t the slope at the inflection point ξ, s the size of the lag domain and $\tilde{q}$ the shape of the curve, as well as, the location of ξ. The model curve is split into three domain parts; a lag domain part ($l \leq s$), an inflection domain part ($s < l \leq o$), and an asymptote domain part ($l > o$). The inflection point ξ is shown for the case $\{A = 1, t = 1, s = 1.5, \tilde{q} \approx 1.0\}$. Bell-shaped curves represent corresponding RDF traces. Note that RDF traces maximize at $l = \xi$.

S4. Modeling of the PDs-VSDs geometrical crossover

We approximated the radial distribution function (RDF) of $N(\mathbf{p}, l_\alpha(\mathbf{p}))$ atoms around $\mathbf{p}$ by

$$G(\mathbf{p}, l_\alpha(\mathbf{p})) = \frac{\Delta N(\mathbf{p}, l_\alpha(\mathbf{p}))}{\frac{4}{3} \cdot \pi \cdot (l_\alpha(\mathbf{p})^3 - (l'_\alpha(\mathbf{p}))^3) \cdot \rho(\mathbf{p})} \text{ with } l'_\alpha(\mathbf{p}) = l_\alpha(\mathbf{p}) - \Delta l_\alpha(\mathbf{p}) \quad \text{(S1)}$$

where $\Delta l_\alpha(\mathbf{p}) = \frac{L(\mathbf{p}) - \bar{R}(\mathbf{p})}{K_\alpha}$ is the thickness of a spherical shell around $\mathbf{p}$, $\Delta N(\mathbf{p}, l_\alpha(\mathbf{p})) = N(\mathbf{p}, l_\alpha(\mathbf{p})) - N(\mathbf{p}, l'_\alpha(\mathbf{p}))$ is the number of atoms found within the spherical shell of thickness $\Delta l_\alpha(\mathbf{p})$ centered at $\mathbf{p}$ and $\rho(\mathbf{p}) = \frac{N_c}{V(\mathbf{p})} = \frac{N_c}{\frac{4}{3} \cdot \pi \cdot (L(\mathbf{p}) - R(\mathbf{p}))^3}$ is the average atom-packing density around $\mathbf{p}$.

Next, we divided all N_c atoms into two groups, one containing only PD atoms, i.e., atoms forming the PDs, and one containing only VSD atoms, i.e., atoms forming the VSDs. The total number of PD atoms is $N_{PD} = 6068$ and they belong to the residue sequence $M130{:}M221$ that forms the structural units

of the S5 domain, the pore turret loop, the P-helix, the selectivity filter, the P2-helix and the S6 domain (see Suppl. Fig. 1 of [2]). On the other hand, the total number of VSD atoms is $N_{VSD} = 8708$ and they belong to the residue sequence $M001$:$G129$ that forms the structural units of the S1N helix, the S1 domain, the S1-S2 loop, the S2 domain, the S2-S3 loop, the S3 domain, the S3-S4 loop, the S4 domain and the S4-S5 linker (see Suppl. Fig. 1 of [2]). Note that $N_c = N_{PD} + N_{VSD}$.

Using equation S1 we approximated the RDFs of the PD and VSD atoms around $\mathbf{p}$ with

$$G_{PD}(\mathbf{p}, l_\alpha(\mathbf{p})) = \frac{\Delta n_{PD}(\mathbf{p}, l_\alpha(\mathbf{p}))}{\frac{4}{3}\cdot\pi\cdot(l_\alpha(\mathbf{p})^3 - (l'_\alpha(\mathbf{p}))^3)\cdot\rho_{PD}(\mathbf{p})} \tag{S2}$$

and

$$G_{VSD}(\mathbf{p}, l_\alpha(\mathbf{p})) = \frac{\Delta n_{VSD}(\mathbf{p}, l_\alpha(\mathbf{p}))}{\frac{4}{3}\cdot\pi\cdot(l_\alpha(\mathbf{p})^3 - (l'_\alpha(\mathbf{p}))^3)\cdot\rho_{VSD}(\mathbf{p})} \tag{S3}$$

, respectively, where $N(\mathbf{p}, l_\alpha(\mathbf{p})) = N_{PD}(\mathbf{p}, l_\alpha(\mathbf{p})) + N_{VSD}(\mathbf{p}, l_\alpha(\mathbf{p}))$, $\rho_{PD}(\mathbf{p}) = \frac{N_{PD}}{V(\mathbf{p})}$ and $\rho_{VSD}(\mathbf{p}) = \frac{N_{VSD}}{V(\mathbf{p})}$.

In order to obtain a relative measure of how the RDF of PD atoms varies with respect to the RDF of VSD atoms, and vice versa, we introduced the equilibrium PDs-VSDs RDF function [1]

$$e(\mathbf{p}, l_\alpha(\mathbf{p})) = sm(G_{PD}(\mathbf{p}, l_\alpha(\mathbf{p})) - G_{VSD}(\mathbf{p}, l_\alpha(\mathbf{p}))) \tag{S4}$$

where $sm(\cdot)$ implements the Nadaraya-Watson kernel regression function with a bandwidth parameter value of $bw = 2$. $e(\mathbf{p}, l_\alpha(\mathbf{p})$ is interpreted as the smoothed probability of finding a PD atom instead of a VSD atom, and vice-versa, at distance $l_\alpha(\mathbf{p})$ from $\mathbf{p}$. Specifically, if $e(\mathbf{p}, l_\alpha(\mathbf{p})) > 0$ ($e(\mathbf{p}, l_\alpha(\mathbf{p})) < 0$) then the smoothed probability of finding a PD atom within the spherical shell of width $\Delta l_\alpha(\mathbf{p})$ is larger than that of finding a VSD (PD) atom. Accordingly, what is of interest here is the sign-change behavior of $e(\mathbf{p}, l_\alpha(\mathbf{p})$ for increasing $l_\alpha(\mathbf{p})$. We investigated it by detecting for every $\mathbf{p} \in Q$ the pair $\{l'_\alpha(\mathbf{p}), l_\alpha(\mathbf{p})\}$ for which the sign-change condition $e(\mathbf{p}, l'_\alpha(\mathbf{p}))\cdot e(\mathbf{p}, l_\alpha(\mathbf{p})) < 0$ is satisfied and calculating the sampling radius

$$\nu(\mathbf{p}) = l'_\alpha(\mathbf{p}) - \frac{e(\mathbf{p}, l'_\alpha(\mathbf{p}))}{e(\mathbf{p}, l_\alpha(\mathbf{p})) - e(\mathbf{p}, l'_\alpha(\mathbf{p}))} \cdot \Delta l_\alpha(\mathbf{p}) \tag{S5}$$

for which $e(\mathbf{p}, l_\alpha(\mathbf{p}))$ changes sign along $l_\alpha(\mathbf{p})$-direction. Equation S5 roughly quantifies the size of the PDs thus indicating the location around the pore of the geometrical crossover from the PDs to the VSDs takes place.

S5. Statistical representation of scalar functions

Let $\langle\cdot\rangle$ be a statistical operator returning the median of the data set upon which it operates. Note that the choice of the median as a statistical measure reflects the fact that no assumption has been made for the distribution of data set values.

Statistical representation of scalar function $f(\mathbf{p}, l_\alpha(\mathbf{p}))$. A statistical representation of the scalar function $f(\mathbf{p}, l_\alpha(\mathbf{p}))$ for a given α was obtained in terms of $\langle f(\mathbf{p}, l_\alpha(\mathbf{p}))\rangle_\alpha$ where the subscript α indicates that the statistical operator acts for a given α, i.e., on the set of values $\Gamma(\alpha) = \{f(\mathbf{p}, l_\alpha(\mathbf{p})) \mid \mathbf{p} \in Q\}$.

Statistical representations of scalar functions $s(\mathbf{p})$, $\nu(\mathbf{p})$, $\xi(\mathbf{p})$ and $o(\mathbf{p})$. Statistical representations of the functions $s(\mathbf{p})$, $\nu(\mathbf{p})$, $\xi(\mathbf{p})$ and $o(\mathbf{p})$ were obtained by the following algorithmic scheme:

Step 1. Let $f(\mathbf{p})$ represent one of the aforementioned scalar functions, then, for every $\mathbf{p}$ find the α indexes for which $|f(\mathbf{p}) - l_\alpha(\mathbf{p})|$ is minimized, i.e., $\alpha_f = \{\alpha \mid \underset{\alpha\in A}{min}(|f(\mathbf{p}) - l_\alpha(\mathbf{p})|)\}$.

Step 2. Calculate the median $\langle\alpha_f\rangle$ of the data set α_f. Note that the minimum and the maximum value contained in α_f is given by $min(\alpha_f)$ and $max(\alpha_f)$.

S6. Scaling behavior of the hydropathic imbalance magnitude along the pore

We scanned all different paths leading from the NavAb's pore axis toward its outer pore surface. The scaling behavior of the HIIS for every path corresponding to a $\mathbf{p}\in Q$ was checked for its power-law property. That was done by linear approximation on $\log[I(\mathbf{p}, l_\alpha(\mathbf{p}))]$ for the lag domain interval, i.e., $l_\alpha(\mathbf{p}) \le s(\mathbf{p})$, and for the pre- and post-inflection phase intervals, i.e., $s(\mathbf{p}) < l_\alpha(\mathbf{p}) \le \xi(\mathbf{p})$ and $l_\alpha(\mathbf{p}) > \xi(\mathbf{p})$, respectively. Accordingly, for each interval we obtained a linear

fitting of the form $\gamma(\mathbf{p})\cdot\log[l_\alpha(\mathbf{p})] + \beta(\mathbf{p})$ where the "goodness" of the fitting was evaluated in terms of the Pearson coefficient, as well as, in terms of the uncertainty in estimation of $\gamma(\mathbf{p})$ retrieved from the mean absolute fitting error (MAFE).

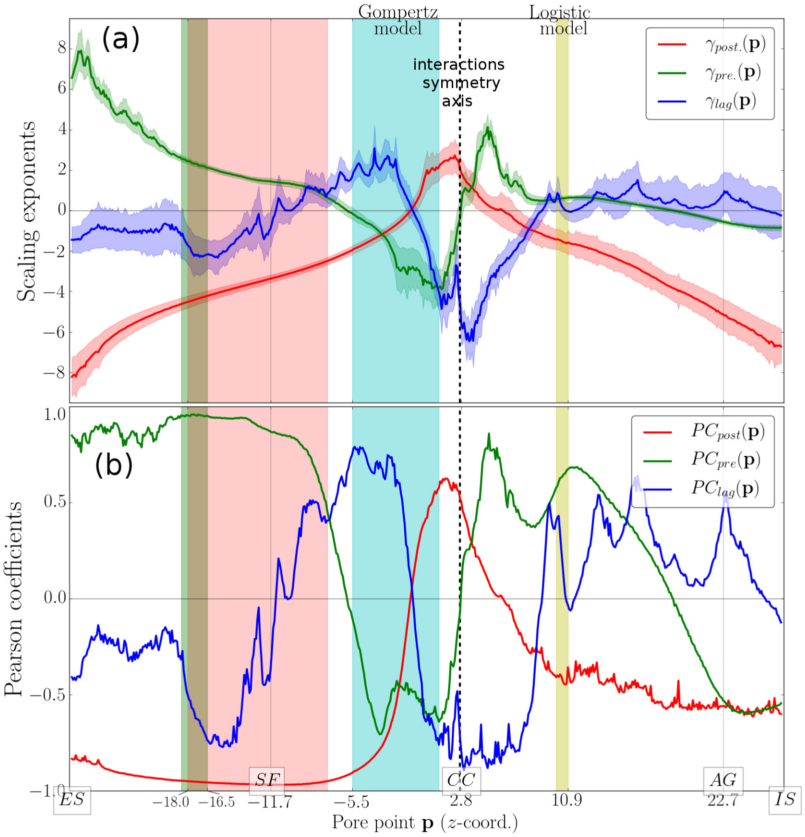

Figure S3. Scaling analysis of the inter-atomic hydropathic interaction strength. (a), Traces of the power-law exponents $\gamma_{lag}(\mathbf{p})$, $\gamma_{pre}(\mathbf{p})$ and $\gamma_{post}(\mathbf{p})$ describing the scaling behavior of the inter-atomic hydropathic interaction strength within lag interval $l_\alpha(\mathbf{p}) \leq s(\mathbf{p})$, a pre-inflection interval $s(\mathbf{p}) < l_\alpha(\mathbf{p})) \leq \xi(\mathbf{p})$ and the post-inflection interval $l_\alpha(\mathbf{p})) > \xi(\mathbf{p})$, respectively, are plotted for every pore point $\mathbf{p} \in Q$. Shaded area around $\gamma_{lag}(\mathbf{p})$, $\gamma_{pre}(\mathbf{p})$ and $\gamma_{post}(\mathbf{p})$ indicate uncertainty in their calculation in terms of the mean absolute fitting error. (b), Traces of the corresponding Pearson coefficients $PC_{lag}(\mathbf{p})$, $PC_{pre}(\mathbf{p})$ and $PC_{post}(\mathbf{p})$ are plotted. Green-shaded

and red-shaded areas indicate pore regions where $|PC_{pre}(\mathbf{p})|$ and $|PC_{post}(\mathbf{p})|$ attain values larger than 0.95, respectively. The intersection of the green-shaded and red-shaded areas the pore region $-18.0 \leq \mathrm{p}_z \leq -16.5$ located at the extracellular side (ES) of the selectivity filter (SF). AG stands for activation gate. IS stands for intracellular side. CC stands for central cavity.

As we demonstrate in Fig. S3, HIIS profiles along $\mathbf{p}$-direction are organized with respect to $\mathrm{p}_z \approx 2.8$ corresponding to the center of the CC, as well as, to the hydropathic topological center of the pore controlling its gating behavior [1]. Pre-inflection hydropathic interactions exhibit an odd pseudo(i.e., non-exact)-symmetric behavior with respect to $\mathrm{p}_z \approx 2.8$ as indicated by the behavior of the $\gamma_{pre}(\mathbf{p})$ trace (see Fig. S3(a)). On the other hand, $\gamma_{lag}(\mathbf{p})$ and $\gamma_{post}(\mathbf{p})$ exhibit an even pseudo(i.e., non-exact)-symmetric behavior with respect to an interactions-symmetry-axis placed at $\mathrm{p}_z \sim 2.8$ perpendicular to the pore axis (see Fig. S3(a)). This symmetry scheme suggests that what is actually differentiating among the IS and the ES is the range of hydropathic interactions within the PDs; both $\gamma_{lag}(\mathbf{p})$ and $\gamma_{post}(\mathbf{p})$ attain negative values toward the IS and the ES but, in striking contrast, $\gamma_{pre}(\mathbf{p})$ exhibits a strong, positive increase at the ES and a weak decrease towards negative values at the IS. The transition to negative values of the triplet $\{\gamma_{lag}(\mathbf{p}), \gamma_{pre}(\mathbf{p}), \gamma_{post}(\mathbf{p})\}$ at the IS appears to initiate at the right of the Logistic model pore region (i.e., for $\mathrm{p}_z > 10.9$). On the other hand, the transition of $\gamma_{pre}(\mathbf{p})$ to a large, positive value initiates on the left of the Gompertz model pore region (i.e., for $\mathrm{p}_z < -5.5$). Taken together, these observations suggest that the range of interactions stabilizing the closed AG is complexly depending on scale and, most likely, stays short while long-range interactions emerge within the PDs toward the ES. Indeed, a clear enhancement of the power-law scaling of $I(\mathbf{p}, l_\alpha(\mathbf{p}))$ occurs on the left of the Gompertz model pore region, i.e., for $\mathrm{p}_z < -5.5$. In particular, $PC_{post}(\mathbf{p})$ attains values smaller than -0.95 for $-18.0 \leq \mathrm{p}_z \leq -7.4$ (see red-shaded area in Fig. S3(a),(b)) so that it minimizes at the SF, i.e., for $\mathrm{p}_z \approx -11.7$, while $PC_{pre}(\mathbf{p})$ attains values higher than 0.95 for $-18.0 \leq \mathrm{p}_z \leq -16.5$ so that it maximizes at the ES of the SF (see green-shaded area in Fig. S3(a),(b)). Accordingly, within the narrow interval $-18.0 \leq \mathrm{p}_z \leq -16.5$ both $|PC_{pre}(\mathbf{p})|$ and $|PC_{post}(\mathbf{p})|$ attain their maximum values while the uncertainty in their calculation remains small (see shaded are around $\gamma_{pre}(\mathbf{p})$ and $\gamma_{post}(\mathbf{p})$ in Fig. S3(a)) allowing for an accurate approximation of HIIS in

terms of

$$I(\mathbf{p}, l_\alpha(\mathbf{p})) = \begin{cases} l_\alpha(\mathbf{p})^{\gamma_{pre}(\mathbf{p})} & \text{for} \;\; s(\mathbf{p}) < l_\alpha(\mathbf{p}) \leq \xi(\mathbf{p}) \\ l_\alpha(\mathbf{p})^{\gamma_{post}(\mathbf{p})} & \text{for} \;\; l_\alpha(\mathbf{p}) > \xi(\mathbf{p}) \end{cases} \tag{S6}$$

The $-18.0 \leq \mathrm{p}_z \leq -16.5$ pore region is directly surrounded by the $M181$ and the $S178$ residue side chains as shown in [1]. The highly-conserved nature of the pore-forming residues around $-18.0 \leq \mathrm{p}_z \leq -16.5$ is shown in Fig. S4 (see also [2]).

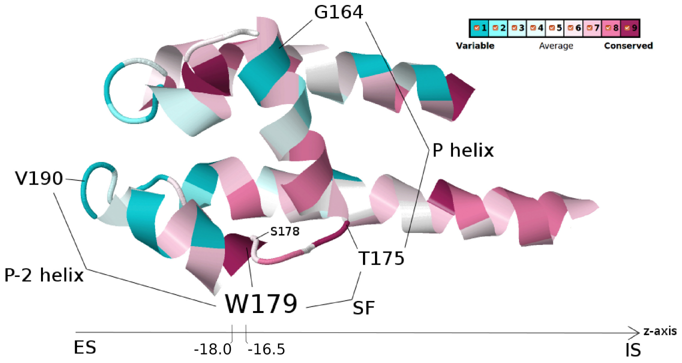

Figure S4. Conservation analysis of a PD domain. Conservation analysis of a single PD domain. The SF, and the P- and P2-helices are annotated. The highest conservation score is attributed to the SF residue complex $T175$-$L176$-$E177$-$S178$-$W179$ scoring an average of 2.4 (out of 4.0) with pore-lining residue conservation scores confined within $[0, 2.0]$. Analysis was performed on the ConSurf Server (http://consurf.tau.ac.il/) according to the algorithmic implementations described in [3, 4].

References

[1] Xenakis, M.N., Kapetis, D., Yang, Y. et al. Cumulative hydropathic topology of a voltage-gated sodium channel at atomic resolution. *Proteins* **88**, 1319-1328, (2020).

[2] Payandeh, J., Scheuer, T., Zheng, N., Catterall, W.A. The crystal structure of a voltage-gated sodium channel. *Nat* **475**, 353-358 (2011).

[3] Landau, M., Mayrose, I., Rosenberg, Y., Glaser, F., Martz, E., Pupko, T., Ben-Tal, N. ConSurf 2005: the projection of evolutionary conservation scores of residues on protein structures. *Nucl Acids Res* **33**, W299-W302 (2005).

[4] Glaser, F., Pupko, T., Paz, I., Bell, R.E., Bechor-Shental, D., Martz, E., Ben-Tal, N. ConSurf: Identification of Functional Regions in Proteins by Surface-Mapping of Phylogenetic Information. *Bioinformatics* **19**, 163-164 (2003)
